# Protective Effect of Diet-Supplemented and Endogenously Produced Omega-3 Fatty Acids against HFD-Induced Colon Inflammation in Mice

**DOI:** 10.3390/foods11142124

**Published:** 2022-07-18

**Authors:** Shalom Sara Thomas, Youn-Soo Cha, Kyung-Ah Kim

**Affiliations:** 1 Department of Food Science and Human Nutrition, Jeonbuk National University, Jeonju 54896, Korea; shalomsarathomas@gmail.com (S.S.T.); cha8@jbnu.ac.kr (Y.-S.C.); 2 Obesity Research Center, Jeonbuk National University, Jeonju 54896, Korea; 3 Department of Food and Nutrition, Chungnam National University, Daejeon 34134, Korea

**Keywords:** alpha-linolenic acid, inflammation, NF-κB, omega-3 fatty acids, vegetable oil

## Abstract

Perilla (*Perilla frutescens*) oil reduces high-fat-diet-induced colon inflammation by suppressing the NF-κB pathway. In the current study, we compared the effect of endogenously produced and externally supplemented omega-3 fatty acids on high-fat-diet-induced colon inflammation. The *fat-1* transgenic mice that endogenously synthesize omega-3 fatty acids were backcrossed with C57BL/6J wild-type mice to obtain transgenic (TR) and wild-type (WT) littermates. Five-week-old male littermates were divided into five groups: two groups fed 10% normal diet (WTLD, TRLD) and three groups fed with a 60% fat high-fat diet (WTHD, TRHD, and WTPO). In the WTPO group, 8% (*w*/*w*) of perilla oil was added. Perilla oil supplemented WT mice and *fat-1* transgenic mice suppressed high-fat-diet-induced body weight and improved serum lipid levels. Furthermore, the WTPO and TRHD groups exhibited increased colon length, lower macroscopic scores, and reduced levels of pro-inflammatory markers and improved epithelial integrity barrier markers. The expression of GPR120 was increased in the WTPO group. Altogether, our results indicated that perilla oil could improve the symptoms of colon inflammation as an alternate omega-3 fatty acid supplement.

## 1. Introduction

Prolonged consumption of a high-fat diet (HFD) results in the accumulation of fat in the body, increasing health risks and causing several health complications such as cardiovascular disease, hypertension, fatty liver, blood pressure, type 2 diabetes, and colitis that contribute to the increasing economic burden in society [1]. Inflammatory reactions resulting from chronic HFD consumption contribute significantly to the initiation of obesity-associated complications. Shifting of the intestinal microbiota toward an increased ratio of *Enterobacteriaceae* owing to a HFD could be the first step toward chronic systemic inflammation. Changes in intestinal microbiota stimulate the Toll-like receptor pathway, that affects the intestinal permeability to endotoxins, increasing its penetration into the circulation [2]. Furthermore, intestinal cells may also be affected by the increased free fatty acid (FFA) levels in the HFD. These elevated levels of endotoxins and FFAs result in the enhanced generation of pro-inflammatory cytokines including tumor necrosis factor (TNF-α), interleukin (IL)-1β, and IL-6 in the gut. Persistence of such condition leads to a continuous flow of lipopolysaccharides (LPS), pro-inflammatory cytokines, and FFA into the systemic circulation, triggering low-grade systemic inflammation [3].

Inflammatory bowel disease (IBD) indicates a set of chronic inflammatory diseases, such as ulcerative colitis (UC) and Crohn’s disease (CD), affecting the gastrointestinal tract. Previously known to be localized only in Western countries, IBD incidence is now increasing in countries of Asia and Africa because of industrialization, changes in dietary patterns, and lifestyle [4]. As the global burden of IBD is anticipated to rise in the years ahead, given the current trend, research and practical interventions to manage or prevent these diseases are needed. Dietary modification involving the supplementation of anti-inflammatory compounds is an effective approach for managing IBD. Particularly, omega-3 fatty acids have been receiving much attention over the past several years because of their improving effects on conditions such as cardiovascular disease, rheumatoid arthritis, and neurodegenerative disorders [5,6,7]. Because of their anti-inflammatory effects, omega-3 fatty acids are also being examined for attenuating IBD. Several studies in experimental animal models have reported that omega-3 fatty acids present in fish oil ameliorate colitis [8,9]. Furthermore, dietary intake of eicosapentaenoic acid (EPA) and docosahexaenoic acid (DHA) has been reported to lower the risk of UC in humans [10]. Surprisingly, the possible effects of plant-derived omega-3 fatty acid-rich oils on IBD have not been as extensively studied as those of fish oil (FO). Perilla oil (PO), extracted from the seeds of *Perilla frutescens*, has a high content of omega-3 fatty acids. It is reported to have anti-inflammatory, anti-atherosclerotic, and lipid-lowering properties in animal models [11,12,13]. Toshiaki et al. reported that PO with various concentrations of vitamin E reduced the levels of leukotriene B4 (LTB4) in a UC mice model [14]. A study conducted on an experimental model of CD showed that PO significantly suppressed the levels of plasma LTB4, and the effect was better than the fish-oil-supplemented group [15]. Considering the regular use of PO in Korean cuisine, we aimed to investigate the possible effects of PO on colon inflammation in different animal models.

The *fat-1* transgenic mice contain the *fat-1* gene, that encodes a desaturase enzyme that can produce omega-3 fatty acids from omega-6 fatty acids within the body. The generation of *fat-1* transgenic mice has facilitated the investigation of the effects of omega-3 fatty acids in different disease conditions by completely avoiding interference from other dietary factors. Some previous studies with *fat-1* transgenic mice have shown that the endogenous omega-3 fatty acids protect against UC and CD [16,17]. In a previous study, we observed that PO exerts protective effects against HFD-induced colon inflammation. The effects were similar to those of FO, which was used as a positive control [18]. In the present study, we evaluated the comparative effect of endogenously produced omega-3 fatty acids in *fat-1* transgenic mice and diet-supplemented omega-3 fatty acids in wild-type (WT) mice against HFD-induced colon inflammation.

## 2. Materials and Methods

### 2.1. Animals and Diet

All experimental procedures carried out were permitted by the Animal Use and Care Committee of Chungnam National University (CNU-01038). Mice were sustained in a temperature-regulated housing, 23 °C ± 1 °C, with light and dark cycles for 12 h each. A set of *fat-1* transgenic male mice were kindly provided by Dr. Byung Hyun Park (Jeonbuk National University Medical School, Jeonju, Korea) [19]. The mice were crossed with C57BL/6J female mice to obtain *fat-1* transgenic and WT littermates. Five-week-old WT littermates were divided into three groups (*n* = 8, total 24): WTLD, WTHD, and WTPO; while transgenic littermates were divided into two groups (*n* = 8, total 16): TRLD and TRHD. A total of 40 mice were used in the study. The animals were divided randomly so that the groups did not have any significant differences in their body weight before starting the experimental diet. The experimental diet given to each group is as follows: WTLD and TRLD—10% fat diet (low-fat diet, LD); WTHD, TRHD, and WTPO—60% fat diet (high-fat diet, HFD).

For the WTPO group, the diet named as HD + PO was made by replacing 25% of lard with edible PO, resulting in a concentration of 8% (*w*/*w*) of PO in the diet. The PO used was cold-pressed, refined oil obtained from a local market. The fatty acid composition of PO used in the study is given in Table 1.

Body weight and feed intake were measured weekly and thrice a week, respectively, throughout the 16 weeks of the experiment. The diet composition is given in Table 2.

Low-fat diet (LD), 10% kcal from fat; high-fat diet (HD), 60% kcal from fat; HD + PO diet, 60% kcal from fat diet containing 8% perilla oil in diet. The sign “-“ indicates that the component is not added to the diet.

### 2.2. Sample Collection

Mice were euthanized following 12 h of fasting. Tissues were collected, frozen immediately, and stored at −72 °C, and some parts of the tissues were fixed in formalin for histological evaluation. After dissection of the colon, edema or ulceration level was recorded as previously reported [20].

### 2.3. Histological Analysis

Longitudinally dissected colon tissue was fixed in 10% formalin, embedded in paraffin, sectioned and stained using hematoxylin and eosin (H&E). Stained slides were visualized using an Axiophot Zeiss Z1 microscope (Carl Zeiss, Gottingen, Germany).

### 2.4. Fecal Bacterial Count

Samples diluted using autoclaved PBS were cultured for 24 h on Desoxycholate agar for *Enterobacteriaceae* and for 48 h on BL agar for *Bifidobacteria* at 37 °C. Agar was obtained from MB cell, Seoul, Korea.

### 2.5. Serum Parameters

Biochemical assays to measure serum triglyceride (TG), total cholesterol (TC), and HDL-C were performed using appropriate kits (ASAN Bio, Seoul, Korea). Pro-inflammatory cytokines, such as IL-1β, IL-6, and TNF-α, in serum and colon, were measured using kits (R&D Systems, Minneapolis, MN, USA). Endotoxin levels in serum were quantified using a Pierce LAL Chromogenic Endotoxin Quantitation Kit (Thermo Fischer Scientific, Rockford, IL, USA).

### 2.6. RT-qPCR and Western Blot

RT-qPCR was used to measure colon-inflammation-related mRNA expression. The purity of RNA was confirmed after extraction using the QIAGEN RNA easy kit (QIAGEN GmbH, Hilden, Germany). Further, using the cDNA synthesized by a cDNA reverse transcription kit (Applied Biosystems, Foster City, CA, USA), qPCR was carried out using the primers presented in Table 3.

For Western blot, colon tissue lysate was obtained using RIPA buffer consisting of protease and phosphatase inhibitors and was quantified by BCA assay. In total, 20 μg of protein were loaded and separated using 10% SDS-PAGE and transferred to a PVDF membrane. The target proteins were detected after incubation with primary and secondary antibodies, followed by detection using an ATTO LuminoGraph II (ATTO, Tokyo, Japan) imaging system.

### 2.7. Statistical Analysis

One-way analysis of variance using SPSS software (version 17.0, SPSS Inc., Chicago, IL, USA) was used to perform statistical analyses. Values with *p* < 0.05 obtained after Duncan’s test were considered statistically significant and are indicated with different superscripts (a, b, and c). Data are expressed as mean ± standard deviation.

## 3. Results

### 3.1. PO-Supplemented WT and fat-1 Transgenic Mice Exhibited Improved Anthropometric Parameters and Serum Lipids

**Figure 1:**
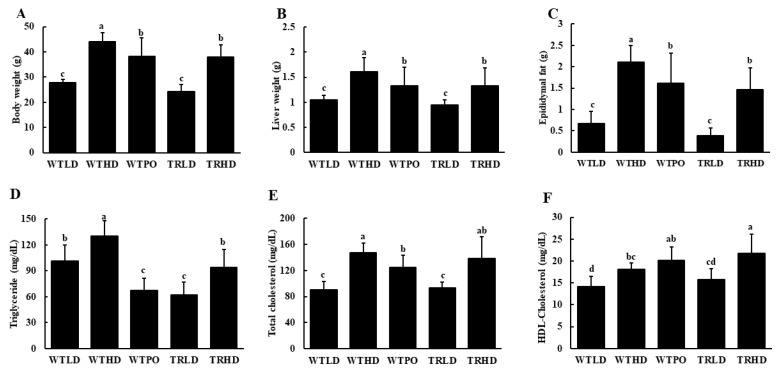
(**A**) Final body weight, (**B**) liver weight, (**C**) epididymal fat weight, (**D**) serum TG, (**E**) serum TC, and (**F**) HDL-C. Mice were divided into five groups (*n* = 8); WTLD and TRLD fed a low-fat diet (LD, 10% kcal from fat); WTHD and TRHD fed a high-fat diet (HD, 60% kcal from fat); WTPO fed an HD + PO diet (60% kcal from fat diet, supplemented with 8% perilla oil in the diet). Values are expressed as mean ± SD, and those with different superscript (a, b, c) letters indicate significance between groups by ANOVA with Duncan’s test at *p* < 0.05.

Consumption of HFD significantly increased body weight in all three HFD-fed groups compared to LD-fed groups (Figure 1A). However, WTPO and TRHD groups showed significantly lower body weights than the WTHD group. The liver and epididymal fat weights showed similar patterns (Figure 1B,C). Compared to the WTHD group, WTPO and TRHD groups had significantly lower organ weights. In the case of LD-fed mice, both wild-type and transgenic mice did not show a difference in body weight and organ weights, although TRLD had slightly lower values. The feed intake was not significant between groups (WTLD—2.83 ± 0.07 ns; WTHD—2.59 ± 0.32 ns; WTPO—2.83 ± 0.37 ns; TRLD—3.18 ± 0.64 ns; TRHD—2.51 ± 0.05 ns).

Analysis of serum lipids showed that PO supplementation in wild-type mice and endogenously produced omega-3 fatty acids in transgenic mice significantly reversed HFD-induced rise in serum TG levels (Figure 1D). The WTHD group exhibited significantly higher TG levels than the other experimental groups. Interestingly, the WTLD and TRHD groups had similar TG levels. Notably, serum TG of the WTPO group was similar to that of the TRLD group. Compared to the WTHD group, WTPO had significantly reduced levels of serum TC, and the TRHD group showed a tendency to reduce TC levels (Figure 1E). The TRHD group had significantly higher levels of HDL-C while the WTPO showed the same increasing tendency, compared to that of WTHD group (Figure 1F).

### 3.2. PO-Supplemented WT and fat-1 Transgenic Mice Had a Longer Colon, Lower Macroscopic Score, and Improved Histological Conditions

**Figure 2:**
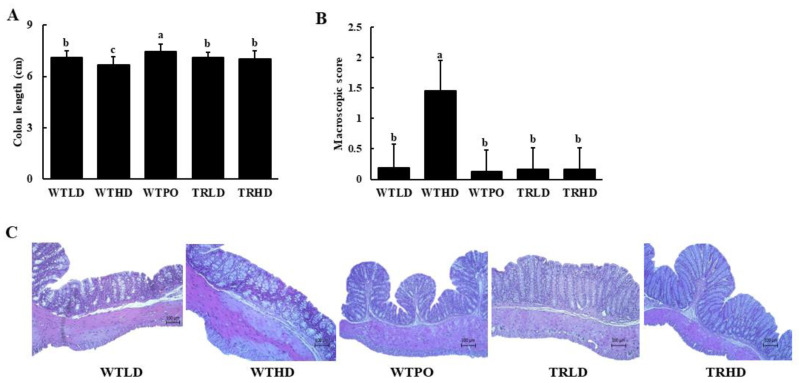
(**A**) Colon length, (**B**) macroscopic score, and (**C**) histological changes. Mice were divided into five groups (*n* = 8); WTLD and TRLD fed a low-fat diet (LD, 10% kcal from fat); WTHD and TRHD fed a high-fat diet (HD, 60% kcal from fat); WTPO fed an HD + PO diet (60% kcal from fat diet, supplemented with 8% perilla oil in the diet). Values are expressed as mean ± SD, and those with different superscript (a, b, c) letters indicate significance between groups by ANOVA with Duncan’s test at *p* < 0.05.

As shown in Figure 2A, HFD intake resulted in a decreased colon length in the WTHD group compared to that in the LD-fed groups. Interestingly, WTPO and TRHD groups showed significantly improved colon lengths than the WTHD group, and the colon length of the WTPO group was significantly longer than that of WTLD, TRLD, and TRHD groups, suggesting the protective effect of PO on colon health. The macroscopic score was significantly increased in the WTHD group, whereas WTPO and TRHD showed significantly lower macroscopic scores (Figure 2B). The H&E staining showed that the endothelia of HFD-fed groups were disrupted (Figure 2C) while WTPO and TRHD showed an improved colon with tightly arranged goblet cells and undisrupted epithelia.

### 3.3. PO-Supplemented WT and fat-1 Transgenic Mice Showed Improved Microbial Population and Decreased Endotoxin Levels in Serum

The composition of intestinal microbiota was altered in the WTHD group, with an increase in the number of *Enterobacteriaceae* and a decrease in the number of *Bifidobacteria* (Table 4). Compared to the WTHD group, all the other groups, including WTLD, TRLD, WTPO, and TRHD, showed a significantly reduced number of *Enterobacteriaceae* and an increased number of *Bifidobacteria*. Interestingly, *Bifidobacteria* colonies were the highest in the TRHD group, followed by the WTPO group. The analysis of endotoxin levels in serum revealed that WTLD, TRLD, WTPO, and TRHD groups had significantly lower levels of serum endotoxin than the WTHD group.

### 3.4. PO-Supplemented WT and fat-1 Transgenic Mice Showed Lower Levels of Pro-Inflammatory Cytokines in Serum and Colon

**Figure 3:**
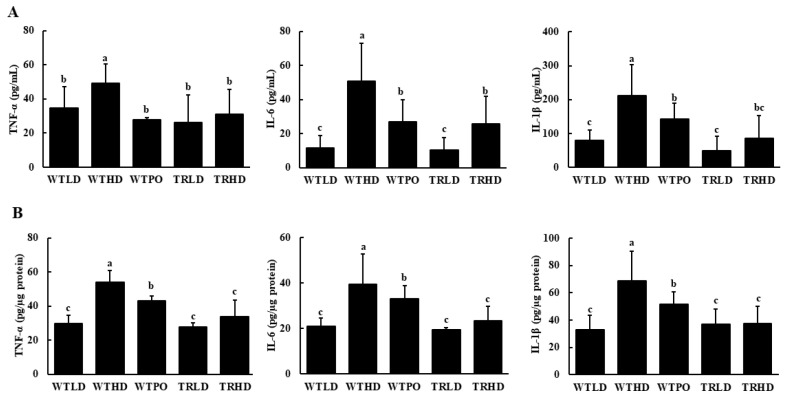
Pro-inflammatory cytokine levels in (**A**) serum and (**B**) colon. Mice were divided into five groups (*n* = 8); WTLD and TRLD fed a low-fat diet (LD, 10% kcal from fat); WTHD and TRHD fed a high-fat diet (HD, 60% kcal from fat); WTPO fed an HD + PO diet (60% kcal from fat diet, supplemented with 8% perilla oil in the diet). Values are expressed as mean ± SD, and those with different superscript (a, b, c) letters indicate significance between groups by ANOVA with Duncan’s test at *p* < 0.05.

As shown in Figure 3, the WTHD group had increased levels of pro-inflammatory cytokines in serum and colon. In serum, WTPO and TRHD groups showed similar levels of TNF-α, IL-6, and IL-1β, which were significantly lower compared to those in the WTHD group (Figure 3A). Similarly, compared with the WTHD group, WTPO and TRHD groups showed significantly reduced levels of TNF-α, IL-6, and IL-1β in the colon (Figure 3B). Furthermore, the TRHD group had significantly lower levels of these cytokines than the WTPO group. The WTLD and TRLD groups had similar levels of pro-inflammatory cytokines, which were lower than the HFD-fed groups.

### 3.5. mRNA and Protein Expression of Inflammatory Markers in the Colon Was Lower in PO-Supplemented WT and fat-1 Transgenic Mice

**Figure 4:**
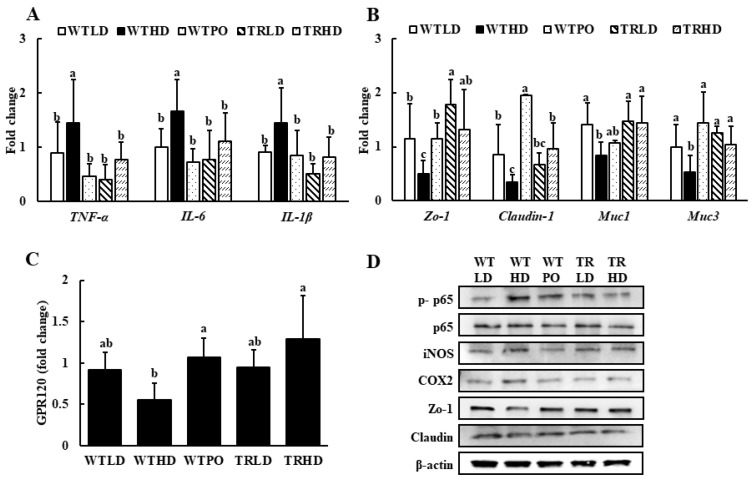
The mRNA expression of (**A**) pro-inflammatory markers, (**B**) epithelial tight junction markers, (**C**) GPR120, and (**D**) protein expression in colon. Mice were divided into five groups (*n* = 8); WTLD and TRLD fed a low-fat diet (LD, 10% kcal from fat); WTHD and TRHD fed a high-fat diet (HD, 60% kcal from fat); WTPO fed an HD + PO diet (60% kcal from fat diet, supplemented with 8% perilla oil in the diet). Values are expressed as mean ± SD, and those with different superscript (a, b, c) letters indicate significance between groups by ANOVA with Duncan’s test at *p* < 0.05.

The mRNA expression levels of TNF-α, IL-6, and IL-1β were significantly upregulated in the WTHD group (Figure 4A). In contrast, WTPO and TRHD groups showed significantly lower levels, similar to that of the LD-fed groups. In addition, WTPO showed enhanced expression of tight junction proteins, such as claudin-1 and Zo-1, and mucins, such as Muc1 and Muc3, compared to the WTHD group (Figure 4B). The TRHD group showed an increasing tendency in claudin-1 expression and significantly higher expression levels of Zo-1, Muc1, and Muc3. Additionally, the mRNA expression of G-protein-coupled receptor 120 (GPR120), a cell membrane receptor stimulated by long-chain fatty acids, was significantly higher in the WTPO and TRHD groups than in the WTHD group (Figure 4C).

As shown in Figure 4D, the protein expression of p-p65, iNOS, and COX2 was lowered in WTPO and TRHD groups compared to the WTHD group. In addition, treatment with PO increased the expression of tight junction protein, Zo-1, and its expression was similar to that of TRLD and TRHD.

## 4. Discussion

There has been an increase in the incidence of IBD worldwide due to various factors such as smoking, infections, antibiotics, high-fat and low-fiber diets. Studies have shown that about 15–40% of IBD patients are obese, and an additional 20–40% are overweight [21]. Inflammatory reactions occurring as a result of a chronic HFD consumption is one of the main mechanisms of initiation of obesity-associated dysfunction of several organs.

Consumption of a HFD for approximately 16 weeks induces gut microbial changes and inflammation in mice [22]. In the present study, we used an HFD-induced colon inflammation mouse model consisting of wild-type C57BL/6J mice and *fat-1* transgenic mice. Our study showed that supplementation with PO, which has an omega-6 to omega-3 fatty acid ratio of 1:4.8, in WT mice and endogenous production of omega-3 fatty acids by *fat-1* mice exerted similar effects in preventing HFD-induced body weight gain, as evidenced by the reduced final body weight and organ weights. Furthermore, the serum lipid-lowering effects were also similar in WTPO and TRHD mice. This suggested that omega-3 fatty acids present in PO could attenuate obesity which was in agreement with some previous reports on PO and *fat-1* transgenic mice [23,24].

After the experimental period, significant differences were observed in the colon lengths between WTHD and WTLD groups. The WT mice fed PO, and transgenic mice that can produce endogenous omega-3 fatty acids prevented colon length shortening, improved macroscopic score, and alleviated serum and colon pro-inflammatory cytokines. The culture of bacteria in the stool showed that both WTPO and TRHD reduced the levels of *Enterobacteriaceae*, which in turn reflected in the reduced levels of endotoxins. A previous study on *fat-1* transgenic mice and *fat-2* transgenic mice (consists of a gene that converts monounsaturated fatty acids to omega-6 fatty acids) showed that the amount of *Enterobacteriaceae* was lower and *Bifidobacteria* was more abundant in *fat-1* transgenic mice than that in *fat-2* transgenic mice [25]. The results obtained in the present study corroborate these results, with *fat-1* transgenic mice showing the highest number of *Bifidobacteria* among all groups, especially when compared with the WTHD group. PO-fed WT mice also showed a similar effect indicating that the ameliorating effect is majorly mediated through omega-3 fatty acids.

It was evident from the colon histology and reduction of pro-inflammatory cytokines in the colon that omega-3 fatty acids suppressed the LPS-induced activation of nuclear factor-kappa B (NF-κB). TNF-α have a significant role in the pathogenesis of IBD, initiating the cytotoxic, apoptotic, and acute-phase reactions and increasing the levels of other pro-inflammatory cytokines, such as IL-1β and IL-6. Supplementation of PO showed lower levels of inflammatory cytokines compared to the WTHD group. Additionally, the suppression of NF-κB activation and the production of iNOS and COX2 in the WTPO and TRHD groups indicated that omega-3 fatty acids downregulated the inflammatory process. A previous study on transgenic mice with TNBS-induced colitis showed that it suppresses NF-κB and increased nuclear factor (erythroid-derived 2)-like 2 (Nrf2) pathways, which was in agreement with our results [17].

Claudin-1 and Zo-1 are major constituents of tight junctions, where epithelial cells are connected, forming a paracellular seal. Mucins are a group of proteins with different functions in the mucus layer barrier of the colon defense system. Three layers of the colon mucosa include (a) glycocalyx formed by the membrane-anchored mucins and epithelial cells, (b) interlinked MUC2 protein layer, and (c) less dense and less viscous outermost layer. Muc1 and Muc3 are membrane-anchored mucins. IBD is characterized by a defective mucosal layer and tight junction proteins [26]. The expression levels of Muc1, Muc3, claudin-1, and Zo-1 were increased in WTPO and *fat-1* transgenic mice, indicating that omega-3 fatty acids improve the intestinal epithelial barrier; thus, preventing the infiltration of bacterial endotoxins into the lamina propria of the intestinal tract and attenuating colon inflammation.

GPR120 is a membrane G-protein-coupled receptor that is known to be activated by omega-3 fatty acids. GPR120 is known to initiate anti-inflammatory responses in cells including macrophages and adipocytes [27]. Activation of GPR120 suppresses the TNF-α or LPS-induced activation of transforming growth factor beta-activated kinase 1 (TAK1), which further inhibits the initiation of the NF-κB signaling pathway. A previous colitis study using an IL-10 knockout mouse reported that GPR120 acts as an anti-inflammatory agent [28]. In our study, the expression of GPR120 was upregulated in WTPO and TRHD compared to the WTHD group, indicating that the improvement effect of omega-3 fatty acids in the colon could be mediated through the stimulation of the GPR120 receptor. 

## 5. Conclusions

PO attenuated HFD-induced colon inflammation in WT mice by attenuating the intestinal barrier protection, suppressing the NF-κB pathway, and decreasing the expression of pro-inflammatory genes, which might be attributed to the activation of GPR120. Comparing the effect of PO provided through the diet to that of the endogenously produced omega-3 fatty acids in the mice, we observed that their anti-inflammatory activities were identical. Through this study, we could establish that PO could be considered as a rich omega-3 fatty acid supplement and its consumption can decrease the omega-6 to omega-3 ratio, therefore, should be encouraged to maintain healthy dietary habits. In addition, clinical trials are needed to validate the daily intake amount of perilla oil in humans.

## Figures and Tables

**Table 1:** Fatty acid composition of PO.

Fatty Acids	Amount Expressed as g/100 g of Perilla Oil
Palmitic acid C16:0	5.8
Palmitoleic acid C16:1	0.07
Margaric acid C17:0	0.06
Stearic acid C18:0	1.8
Linoleic acid C18:2	12.3
α-linolenic acid C18:3	59.7
Arachidic acid C20:0	0.1
Gadoleic acid C20:1	0.2
Arachidonic acid C20:4	0
Docosahexaenoic acid C22:6	0
Saturated fatty acids	7.8
Monounsaturated fatty acids	14.6
Omega-6 fatty acids	12.3
Omega-3 fatty acids	59.7
Omega-6 to omega-3	1:4.8

**Table 2:** Ingredients of the experimental diet.

Ingredient (g)	LD	HD	HD + PO Diet
Corn starch	506.2	0	0
Maltodextrin	125	125	125
Cellulose	50	50	50
Sucrose	68.8	68.8	68.8
Casein	200	200	200
Calcium carbonate	5.5	5.5	5.5
Dicalcium phosphate	13	13	13
Potassium citrate	16.5	16.5	16.5
Vitamin mix	10	10	10
Mineral mix	10	10	10
L-cysteine	3	3	3
Choline bitartrate	2	2	2
Soybean oil	25	25	25
Lard	20	245	182
Perilla oil	-	-	63
Total fat	405	2430	2430
Saturated fat	11.5	101.5	81.24
Omega-6 fatty acids	15.95	40.7	41.59
Omega-3 fatty acids	3.25	3.25	40.86
Omega-6 to omega-3	4.9:1	12.5:1	1:1
Total (g)	1055	773.8	773.8

**Table 3:** Primer list for qPCR.

Gene Name	Primers	Sequence (5′–3′)
TNF-α	F	ACG GCA TGG ATC TCA AAG AC
	R	GTG GGT GAG GAG CAC GTA GT
IL-6	F	AAC GAT GAT GCA CTT GCA GA
	R	GAG CAT TGG AAA TTG GGG TA
IL-1β	F	GAC CTT CCA GGA TGA GGA CA
	R	AGC TCA TAT GGG TCC GAC AG
Claudin-1	F	TCT ACG AGG GAC TGT GGA TG
	R	TCA GAT TCA GCA AGG AGT CG
Zo-1	F	ACC CGA AAC TGA TGC TGT GGA TAG
	R	AAA TGG CCG GGC AGA ACT TGT GTA
Muc1	F	CTG TTC ACC ACC ACC ATG AC
	R	CTT GGA AGG GCA AGA AAA CC
Muc3	F	CCA CCA CTG TTG AAG TCA CAA
	R	CAG AAC CCT CCG TTC ATA CAA
β-actin	F	AGC CTT CCT TCT TGG GTA TGG
	R	CAC TTG CGG TGC ACG ATG GAG

**Table 4:** Fecal count of *Enterobacteriaceae*, *Bifidobacteria*, and endotoxin levels in serum.

	WTLD	WTHD	WTPO	TRLD	TRHD
*Enterobacteriaceae* (CFU/10^1^ X)	5.75 ± 2.62 ^b^	116.00 ± 54.00 ^a^	3.25 ± 0.50 ^b^	2.00 ± 1.73 ^b^	9.00 ± 5.47 ^b^
*Bifidobacteria* (CFU/10^6^ X)	142.25 ± 99.37 ^b^	22.71 ± 9.65 ^c^	143.00 ± 48.68 ^b^	185.66 ± 56.74 ^a,b^	266.25 ± 93.31 ^a^
Endotoxin (EU/mL)	2.04 ± 0.11 ^b^	2.40 ± 0.32 ^a^	2.03 ± 0.12 ^b^	1.99 ± 0.27 ^b^	2.05 ± 0.23 ^b^

Mice were divided into five groups (*n* = 8); WTLD and TRLD fed a low-fat diet (LD, 10% kcal from fat); WTHD and TRHD fed a high-fat diet (HD, 60% kcal from fat); WTPO fed an HD + PO diet (60% kcal from fat diet, supplemented with 8% perilla oil in the diet). Values are expressed as mean ± SD, and those with different superscript (a, b, c) letters indicate significance between groups by ANOVA with Duncan’s test at *p* < 0.05.

## Data Availability

Data can be made available upon request to the corresponding author.
